# African swine fever virus infection of porcine peripheral blood monocyte-derived macrophages induces the formation of tunneling nanotube-connected large vesicle-like cell segments: a potential mechanism for intercellular ASFV trafficking

**DOI:** 10.1186/s13567-025-01582-0

**Published:** 2025-07-10

**Authors:** Brecht Droesbeke, Nadège Balmelle, Hans J. Nauwynck, Herman Favoreel, Marylène Tignon

**Affiliations:** 1 Department Infectious Diseases in Animals, Sciensano, Service Viral Re-emerging, Enzootic and Bee Diseases, Groeselenbergstraat 99, 1180 Brussels, Belgium; 2 Department of Translational Physiology, Infectiology and Public Health, Faculty of Veterinary Medicine, Ghent University, Salisburylaan 133, 9820 Merelbeke, Belgium

**Keywords:** African swine fever virus, tunneling nanotubes, cytopathic effect

## Abstract

African swine fever (ASF) is a highly fatal viral disease in pigs, with mortality rates that can reach 100%. The causative agent, African swine fever virus (ASFV), primarily targets cells of the mononuclear phagocytic system (MPS), particularly monocyte-derived macrophages (MDMs). Despite the severity of the disease, there are currently no effective antiviral treatments available in Europe. A significant barrier to therapeutic development is the limited understanding of how ASFV interacts with its primary target cells. A deeper understanding of the morphological changes induced by ASFV in infected cells is crucial to this effort. To address this knowledge gap, we used conventional and confocal immunofluorescence microscopy, as well as transmission electron microscopy, to investigate ASFV-infected primary MDMs. Our analysis revealed that ASFV infection leads to the formation of large cellular protrusions, which are characterized by vesicle-shaped cellular segments (CSs) at their tips. These protrusions contain all major cytoskeletal components, showing characteristics similar to those of tunneling nanotubes (TNTs). In 84.93% of the cases, the nucleus remained in the cell body (CB) near the viral factory. In the remaining cases, the nucleus was found within these CSs, whereas the viral factory was present in the CB. Additionally, 57.6% of the cells were in contact with the CS and distant cells, suggesting a potential mechanism for ASFV transmission. These findings suggest that ASFV induces cellular segmentation linked by TNT-like structures. Further research is needed to better understand the biogenesis and functional significance of these segmented cells, which could inform future strategies for combating ASFV.

**Supplementary Information:**

The online version contains supplementary material available at 10.1186/s13567-025-01582-0.

## Introduction

Since the discovery of African swine fever (ASF) more than a century ago [1], the disease has spread globally, causing significant economic losses and trade disruptions, as well as raising severe concerns for pig health and welfare. The etiologic agent African swine fever virus (ASFV) is the only known DNA arbovirus and the sole member of the *Asfarviridae* family [2]. It is an enveloped, nucleocytoplasmic large double-stranded DNA virus (NCLDV) comprising between 150 and 167 open reading frames (ORFs) [3]. The genome size varies between 170 and 193 kilo base pairs (kbp) because of the gain or loss of ORFs in the left and right variable regions of the genome [4]. The ASFV virion is notable for its intricate, multilayered architecture. It is composed of several structural layers, including two distinct lipoprotein membranes and two icosahedral protein capsids [5]. The mature extracellular ASFV particle includes the following components: an outer envelope acquired from the host cell membrane during budding, an outer capsid, an inner envelope, an inner capsid or core shell and a nucleoid, which houses the viral genome [5]. Interestingly, intracellular virions that lack the outer membrane are still capable of causing infection [6]. Historically, ASFV strains have been classified into 23 different genotypes (I-XXIII) on the basis of comparative analysis of the C-terminal end of the B646L gene, which encodes the major capsid protein p72 [7, 8]. More recently, a reduction to six genotypes on the basis of the predicted protein sequence of the capsid protein has been proposed [9]. The same research group subsequently adapted its classification of all annotated genomes into seven distinct biotypes, including a specific biotype for recently discovered recombinants of genotypes I and II [10], on the basis of the prediction of the full ASFV proteome. 

The mononuclear phagocytic system (MPS) is the principal site of replication for ASFV [11]. More specifically, monocytes, MDMs and tissue-resident macrophages are the primary target cells for ASFV replication [11, 12]. These cells are versatile immune cells that play critical roles in maintaining tissue homeostasis, modulating inflammation and supporting tissue regeneration [13]. They are highly adaptable and respond to environmental cues and metabolic states to perform a range of functions essential for health and disease management. This adaptability allows macrophages to switch between pro-inflammatory and anti-inflammatory states [14], influencing various physiological and pathological processes. 

To establish a productive infection, ASFV must gain access to the cytosolic compartment of these target cells. After internalisation, ASFV is transported along microtubules to the perinuclear area, where replication and assembly of new virions occur at a specialized assembly site, termed the viral factory (VF) [15]. The entry mechanisms exploited by ASFV remain poorly understood. Early studies provided evidence for the uptake of extracellular ASFV via clathrin-mediated endocytosis [16, 17] or saturable receptor-mediated endocytosis. In the latter, a modulatory role for CD163 and CD169 [18, 19], two scavenging membrane receptors expressed on subsets of porcine tissue-resident macrophages [20], has been proposed. Furthermore, macropinocytosis has been suggested as an additional entry mechanism [21]. Finally, different forms of phagocytosis may also contribute to ASFV uptake [22]. Chen et al. [23] demonstrated that ASFV exploits a form of apoptotic mimicry, with phosphatidylserine (PS) acting as a ligand for AXL, a tyrosine kinase receptor present on porcine alveolar macrophages. In addition to AXL, other receptors present on porcine macrophages are involved in the binding and engulfment of PS-labelled targets [24], providing potential routes for the uptake of the virus. PS is present on the membrane of ASFV-infected apoptotic cells and, consequently, on the envelope of extracellular virions following budding [25]. More recently, Gao et al. reported that apoptotic bodies released from apoptotic ASFV-infected porcine alveolar macrophages (PAMs) can be phagocytosed (via efferocytosis), leading to successful secondary infection of bystander PAMs. Apoptotic bodies are a form of extracellular vesicle (EV), which are membrane-bound vesicles secreted into the extracellular space [26]. EVs encompass a broad range of structures, loosely defined by their biogenesis, biochemical properties, and phenotypical characteristics [27]. These observations suggest that the method of ASFV uptake in secondary cells is inevitably dependent on how the ASFV exits the primary virus-producing cell and reaches the secondary cell for replication. 

The release of ASFV via apoptotic bodies suggests that the virus does not rely fully on virus-controlled budding mechanisms to achieve replication in distant cells. Instead, ASFV leverages a physiological intercellular communication mechanism to facilitate its release and dissemination [28]. Intercellular communication is essential for growth, development, differentiation, tissue and organ formation, and homeostasis [29]. These mechanisms are broadly classified into classical and nonclassical communication [30]. Both forms are exploited by viruses to increase their ability to spread to distant cells. Classical communication occurs between neighbouring cells via direct contact or between distant cells via receptor‒ligand interactions. For example, direct contact-driven viral spread is observed with human immunodeficiency virus type 1 (HIV-1), where virological synapses form between infected and uninfected T lymphocytes or between a dendritic cell and a T lymphocyte [31]. Receptor‒ligand interactions allow cells to detect pathogens by recognizing pathogen-associated molecular patterns, which induce the production of type I interferons, cytokines, and chemokines. These mediators bind to receptors on adjacent cells, restricting viral entry and/or replication [32]. Increasing evidence suggests that viruses exploit these intercellular communication methods to prepare neighbouring cells for subsequent infection [33].

Nonclassical communication mechanisms, such as extracellular vesicles (EVs) and direct contact through membrane protrusions, are particularly advantageous for viruses [27, 29, 30]. These mechanisms allow viral spread without leaving the cytosolic environment of the infected cell, enabling the virus to reach distant cells for replication while being protected from immune responses and harsh extracellular conditions. This enhances viral stability and longevity [34, 35]. Moreover, these modes of spread enable collective transmission of viral genomes, increasing the likelihood of successful infection [36]. Furthermore, these mechanisms facilitate receptor-independent viral entry, broadening the range of host cells that can be infected and potentially enabling cross-species transmission [35]. For ASFV, large EVs are needed because of the size of the infectious virion [6].

Like those of EVs, the classification of membrane protrusions involved in intercellular communication is loosely defined, often on the basis of membrane topology and/or cytoskeletal composition, leading to inconsistent terminology across studies [37]. Among the most recognized cellular protrusions involved in intercellular communication are tunneling nanotubes (TNTs) [37]. TNTs are thin, straight membrane tethers composed of filamentous actin (F-actin) as the main cytoskeletal backbone. They hover above the substrate and form physical connections between distant cellular compartments, spanning multiple cell bodies [38]. Under physiological conditions, TNTs facilitate the intercellular transfer of diverse cargo, including proteins, organelles, receptors, and nucleic acids [39]. Given the significant advantages of this mode of spread, it is unsurprising that a wide array of viruses, e.g., pseudorabies virus (PRV) and porcine reproductive and respiratory disease virus (PRRSV), exploit TNTs for intercellular dissemination [40–42].

Recent developments in this field suggest that the distinction between EVs and membrane protrusions is becoming increasingly blurred. In some cases, membrane protrusions are implicated in the biogenesis of EVs [37, 43]. This broadens the concept of nonclassical communication mechanisms and highlights the limitations of classifying cellular communication systems. These findings also open new research areas for studying the potential role of these genes in pathogen spread.

In this study, we provide a microscopic description of ASFV-induced formation of vesicle-shaped cell segments connected to the remaining cell body via TNT-like projections in infected MDMs in vitro. We quantified the prevalence of these segmented cells over the course of infection and analysed their ability to reach distant cells. Finally, we characterized these structures with respect to their subcellular contents and cytoskeletal composition.

## Materials and methods

### Virus and cells

A sixth passage of the virulent genotype II ASFV Belgium 2018/01 (BE18), which was isolated from the spleen of a dead wild boar found in Etalle (Belgium) in 2018, was used for infection of MDMs [44]. To validate the presence of ASFV-segmented cells in MDMs infected with other ASFV isolates, MDMs were additionally infected with a third passage of the moderately virulent genotype II Est15/WB-Valga-6 (Est) [45], a third passage of the virulent genotype I E70 (E70) [46] and a fourth passage of the attenuated genotype I NH/P68 (NHV) [47]. All virus stocks were propagated in swine peripheral blood mononuclear cells (PBMCs).

For the isolation of MDMs, ± 500 mL of venous blood from three conventional crossbred pigs (Piétrain × hybrid sow) was aseptically collected at the slaughterhouse in sterilized glass bottles containing 15 units/mL heparin (Sigma) dissolved in 40 mL of phosphate-buffered saline (PBS). The blood was further processed within two hours after collection at the slaughterhouse. Upon arrival at the laboratory, the blood was centrifuged for 10 min at 800 × *g* to collect the leukocyte fraction. Negativity for ASFV, PRRSV, PRV and swine influenza (H5N1) was confirmed by real-time quantitative polymerase chain reaction (RT‒qPCR). Afterwards, the leukocyte fraction was diluted with PBS (1:1), and 10 mL was layered on a 15 mL Ficoll‒Paque density gradient (Sigma) in 50 mL Sepmate tubes (Stemcell). The tubes were centrifuged for 10 min at 1200 × *g* to isolate the peripheral blood mononuclear cells (PBMCs). Afterwards, the PBMCs were seeded in T-175 cell culture flasks (Corning) in RPMI + GlutaMAX (Thermo Fisher), supplemented with 1% penicillin (Kela), 1% gentamycin (Gibco), and 1% amphotericin B (Gibco), and incubated for 1.5 h at 37 °C in a 5% CO_2_ atmosphere. After incubation, the medium was discarded, and the flasks were washed three times with heated Hanks’ balanced salt solution (HBSS) to enrich the adhering monocytes. The remaining cells were incubated overnight at 37 °C with 5% CO_2_ in PBMC seeding medium containing RPMI + GlutaMAX supplemented with the same antimicrobial additions and 10% fetal calf serum (FCS) (Gibco). Twenty-four hours later, the adhering cells were detached as described previously [48]. After detachment, the cells were counted with trypan blue exclusion dye in a Luna automated cell counter (Logosbio) and suspended at a concentration of 10^7^ cells/mL in RPMI + GlutaMAX supplemented with 30% FCS and 10% DMSO for subsequent cryopreservation.

After thawing, the cells were resuspended in PBMC seeding medium supplemented with 50 ng/mL recombinant human macrophage-colony stimulating factor (hM-CSF) (Peprotech) at a density of 10^6^ cells/mL. Next, the cells were seeded at a density of 5 × 10^5^ cells/well in a 48-well plate (Greiner) with 8 mm NO. 1 glass coverslips (Epredia). After seeding, the cells were matured toward the MDMs for 96 h, as described previously [49].

### Virus inoculation and sample collection

The MDMs were infected with BE18 at a multiplicity of infection (MOI) of 1. After 1.5 h of incubation at 37 °C in the presence of 5% CO_2_, the inoculum was removed, and the wells were rinsed twice with 1 mL of HBSS. Afterward, 0.5 mL of fresh PBMC seeding medium was added. At 1.5, 9, 15, and 21 h post-infection (hpi), 200 µL of the cell culture supernatants were collected and mixed with 180 µL of ATL buffer (Qiagen) for further determination of the concentration of viral DNA copies via RT‒qPCR. An additional volume (200 µL) of each supernatant was collected for virus titration and stored at −80 °C until analysis. After this, the MDMs were fixed with 6% paraformaldehyde (PFA) in PBS and gently added to the remaining medium to reach a final concentration of 3% PFA for 10 min at room temperature for subsequent immunofluorescence staining.

### Real-time qPCR

DNA was extracted using an automated magnetic bead-based extraction method with an Indimag 48 (Indical Bioscience) and an Indimag pathogen extraction kit following the manufacturer’s instructions. Next, a duplex qPCR assay targeting the viral p72 protein sequence and cellular beta-actin (reference gene) was performed. qPCR was conducted on a LightCycler 480 thermocycler (Roche) according to the method described in [50].

### Virus titration and detection using the immunoperoxidase test (IPT)

The supernatants were titrated on porcine alveolar macrophages (PAMs), and viral detection was performed using an immunoperoxidase test (IPT). Briefly, 24 h prior to inoculation, PAMs (10^5^ cells/well) were seeded into 96-well cell culture plates in PBMC seeding medium supplemented with 1% nonessential amino acids (Gibco) and 1% sodium pyruvate (Sigma) and incubated at 37 °C with 5% CO_2_. The cells were then inoculated with tenfold serial dilutions of the samples in RPMI medium supplemented with GlutaMAX and further incubated under the same conditions. At 72 h post-inoculation, the cells were fixed by first discarding the supernatants and allowing the plates to air dry in an oven at 37 °C for 30 min, followed by heat fixation at 80 °C for 1 h. After fixation, the plates were stored at 4 °C until further processing.

For the detection of infected cells, IPT staining was performed as previously described [51], with the following modifications: after the blocking step, the wells were incubated with a 1:50 dilution of homemade anti-ASFV polyclonal swine serum in blocking buffer. Detection was carried out using protein A conjugated to horseradish peroxidase (Sigma). The stained cells were visualized using a light microscope. Viral titres were calculated as the log_10_ of the 50% tissue culture infectious dose (TCID_50_) per mL using the method described in [52].

### Transmission electron microscopy

One million monocytes were seeded on glass coverslips in 24-well plates and allowed to mature for 4 days in 1 mL of PBMC seeding medium supplemented with 50 ng/mL hM-CSF. After this, the cells were infected with ASFV BE18 (MOI = 1) for 1.5 h. Next, the inoculum was aspirated, the cells were rinsed twice with 1 mL of HBSS, and 1 mL of fresh PBMC medium was added. At 18 hpi, the cells were fixed with 2.5% freshly prepared glutaraldehyde (Electron Microscopy Sciences) for 25 min at 37 °C, followed by fixation with freshly added 2.5% glutaraldehyde for 40 min at room temperature. Next, the cells were washed four times with 0.1 M cacodylate buffer. A post-fixation step was performed by incubating the cells with a 1% osmium tetroxide solution. The cells were then washed three times with ultrapure water, after which a contrasting step was performed by incubation with 1% uranyl acetate for 1 h to improve cell preservation and labelling post-embedding. Next, the samples were dehydrated using a series of graded alcohol concentrations (7%, 15%, 30%, 50%, 70%, 80%, 90%, 2 × absolute) for 10 min each. The dehydrated samples were embedded in Spurr’s resin (Electron Microscopy Sciences). Ultrathin (70-nm) sections were cut with an ultramicrotome (EM [UC6]; Leica). The sections were observed using a JEOL JEM-1010 transmission electron microscope.

### Immunofluorescence microscopy and dyes

After fixation with 3% freshly prepared PFA in PBS, the cells were permeabilized with 0.1% Triton-X 100 for 10 min at room temperature. Immunostaining was performed by incubation for 1 h at 37 °C with mouse monoclonal antibodies or rabbit polyclonal antibodies in blocking buffer containing 10% normal goat serum (NGS). Simultaneously, an isotype antibody against the irrelevant PRV (clone 13D12, kindly provided by the laboratory of Prof. Hans Nauwynck) was used in the same dilution and blocking buffer, confirming the absence of nonspecific binding of the secondary antibody used to label ASFV anti-p72 [53]. The cells were subsequently incubated with compatible secondary antibodies and phalloidin conjugated with fluorescent dyes for 1 h at 37 °C.

To visualize microtubules, the same protocol was followed, except that the cells were rinsed and fixed in 3% PFA and dissolved in cytoskeleton-stabilizing buffer, as described previously [42]. Afterwards, the cells were incubated for 10 min in Hoechst 33342 to identify viral and cellular DNA. The list of antibodies and reagents used in this study is summarized in Table 1. After the staining procedure, the coverslips were mounted with Vectashield mounting medium (Vector Laboratories), fixed with nail polish on an object glass (Epredia), and visualized with an ECLIPSE Ts2R-FL inverted microscope (Nikon) with a 40 × objective or a Zeiss Axio Imager 2 confocal laser scanning microscope equipped with a 63 × oil immersion objective. Images were analysed with Nikon Imaging Software (NIS) Elements Advanced Research (Ar) software (Nikon).

**Table 1:** **Reagents used for fluorescence microscopy**

Target primary antibodies	Clone	Isotype	Working dilution	Supplier
CD172a	BL1H7	Mouse IgG1	1/50	Bio-Rad
CD163	2A10	Mouse IgG1	1/200	Bio-Rad
ASFV major capsid protein p72	1BC11	Mouse IgG1	1/100	Ingenasa
Alpha-tubulin	EP1332Y	Rabbit polyclonal IgG	1/200	Abcam
Vimentin	VI10	Mouse IgM	1/50	Invitrogen

Secondary antibodies (conjugate)	Host/species reactivity	Isotype	Working dilution	Supplier
Cy5	goat anti mouse	IgG1	1/300	Abcam
FITC	goat anti mouse	IgG1	1/200	Abcam
Alexa Fluor 594	goat anti-mouse	IgGM	1/200	Invitrogen
Alexa Fluor 594	goat anti rabbit	IgG polyclonal	1/200	Abcam

Conjugate	Cellular target	Reagent	Working dilution	Supplier
iFluor 594	F-actin	Phalloidin	1/500	Abcam
iFluor 488	F-actin	Phalloidin	1/500	Abcam
inherent fluorescence	DNA	Hoechst 33342	1/1000	Invitrogen

#### Purity of the isolated cells

The purity of the cells prior to infection was analysed via IF microscopy. The cells were stained for the presence of the general porcine myeloid cell marker CD172a and, second, for CD163, a marker that is more specific to monocyte–macrophages [20]. Five random pictures were taken per replicate with an ECLIPSE Ts2R-FL inverted microscope (Nikon) equipped with a 40 × objective. The percentage of membrane marker-positive cells was determined by calculating the fraction of marker-positive cells relative to the total number of cells visualized by staining the nuclei with Hoechst 33342. A total of 95.96% (± 1.82) of the cells were CD172a positive, and 93.66% (± 4.13) of the isolated cells expressed CD163.

#### Percentage of ASFV-infected cells

At the indicated time points post infection, the fraction of ASFV-positive cells was quantified by IF microscopy. The cells were stained for the presence of the ASFV p72 protein with a monoclonal antibody, the cell nuclei were stained with Hoechst 33342, and F-actin was stained with phalloidin-AF 594 to visualize the cell morphology. The percentage of ASFV p72-positive cells was determined by calculating the fraction of p72-positive cells relative to the total number of cells visualized by staining the nuclei with Hoechst 33342.

#### Quantification and phenotyping of segmented cells

At 1.5, 9, 15, and 21 hpi, 600 cells per time point were scored by visual observation of multiple low-power fields directly at the microscope. When present, p72-positive cells were considered for observation. The cellular morphology was revealed by staining the actin cytoskeleton with phalloidin-iFluor 594, concomitant with the presence of the viral p72 antigen with a monoclonal mouse antibody and the nucleus with Hoechst 33342. Cells with straight F-actin projections longer than one cell body in length were quantified. In the case of distant cell contact by a straight actin protrusion between two nucleated cell bodies, only one of the two cell bodies was counted as a positive cell with a TNT-like projection.

A segmented cell in the ASFV-infected group is defined as a cell divided into two or more cell segments linked to each other by thin and straight F-actin-containing tethers that are longer than one cell body (> 10 µm) in distance. At 1.5 hpi, 600 cells per condition were similarly scored because of the absence of p72 positivity in the ASFV-infected MDMs. In the next step, 300 p72-positive segmented cells were scored for the presence or absence of contact with distant cells with the proximal tip of the projection and the presence or absence of the cell nucleus in the cell segment (CS) after image acquisition. In these images, the length of the projections was also calculated with NIS Elements imaging software by manually drawing a line starting from the base of the projection at the cell body (CB) towards the base of the CS.

#### Presence of cytoskeletal components in segmented cells

A total of 180 segmented cells per biological replicate were scored for the presence of all three cytoskeletal components by concomitant visualization of the actin cytoskeleton, viral p72, the nucleus, and vimentin or alpha-tubulin after image acquisition. To determine the mean fluorescence intensity (MFI) signal of F-actin at the CS compared with the MFI of F-actin at the remaining CB, the surface for measurement was manually determined by drawing a line along the circumference of both cell fragments after confocal image acquisition. The MFI in these areas was measured using NIS Elements Ar imaging software.

### Statistical analysis

Every condition was tested in technical triplicates. The data are presented as the mean ± standard deviation (SD) from three independent experiments. Statistical analysis was performed using Prism 9 (GraphPad). Student’s one-tailed paired t test was used to compare the numbers of TNT-like projections, p72 values, and F-actin MFI. A value of *p* < 0.05 was considered significant.

## Results

### ASFV-infected MDMs display an increased number of TNT-like projections

Monocytes were isolated from blood and further matured in vitro in the presence of hM-CSF to increase their adhesion properties. According to Singleton et al. [49], hM-CSF, as a stimulating factor, does not activate monocytes during their maturation process. For this reason, and owing to the enhanced and self-induced adhesive properties of stimulated monocytes, we chose to perform all our experiments with monocytes matured in the presence of hM-CSF.

**Figure 1:**
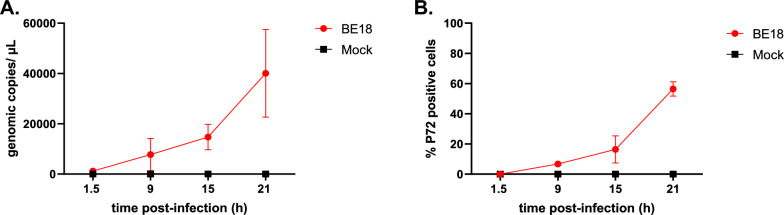
**Infection of monocyte-derived macrophages with ASFV BE18 (MOI = 1). A** At the indicated time points, the supernatants were collected, and the ASFV copy number was determined by RT‒qPCR. **B** Cells were fixed in 3% PFA, and the percentage of infected cells was determined by IF microscopy.

ASFV infection of MDMs and subsequent replication were confirmed by increasing concentrations of ASFV DNA in the supernatants, as measured by RT‒qPCR, from 9 hpi until the end of the experiment (Figure 1A). Furthermore, the titration of infectious virus in the supernatants was positively correlated with increasing ASFV DNA copy numbers in a second experiment conducted under identical conditions, including in PAMs from the same animals, suggesting that the newly produced virus was infectious (Additional file 1). Next, the fraction of p72-positive cells determined by IF microscopy followed a similar pattern, increasing from 9 hpi through the final sampling time point (Figure 1B). Mock-inoculated MDMs remained negative with all the detection methods.

**Figure 2:**
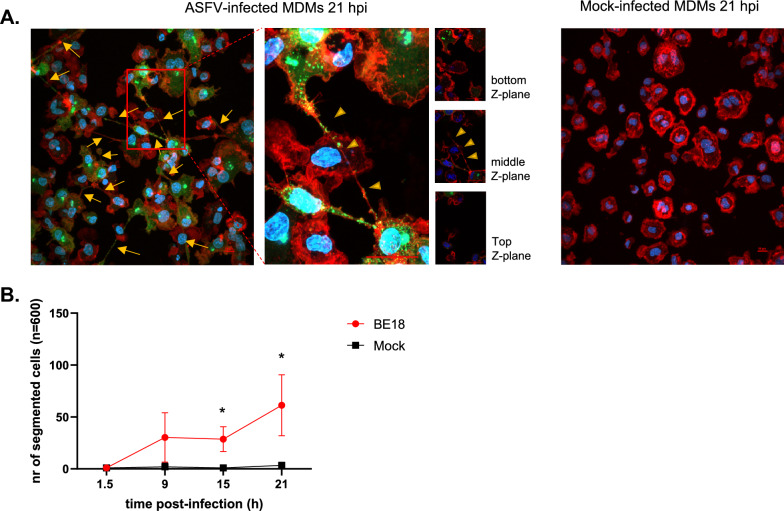
**ASFV infection leads to enhanced formation of TNT-like projections**. **A** Representative images of ASFV- or mock-infected cells at 21 hpi. The cells were stained for the presence of viral p72 (green) with a mouse monoclonal antibody (1BC11), F-actin (red) with Phalloidin-iFluor 594, and DNA with Hoechst 33342. Long, straight F-actin protrusions (yellow arrows) were observed in a fraction of the p72-positive cells. A close-up view of ASFV-infected MDMs reveals a TNT-like projection extending across the cell body of a neighbouring MDM at the apical side (middle z-plane, yellow arrowheads). Scale bar: 10 µm. **B** At 1.5, 9, 15, and 21 hpi, 600 mock-infected and 600 p72-positive cells were scored for the presence of one or more straight F-actin-containing cellular protrusions after immunofluorescence staining of the nuclei, actin cytoskeleton, and viral p72 antigen. Significantly more ASFV-infected cells presented with straight F-actin protrusions at 15 hpi (*p* < 0.01) and 21 hpi (*p* < 0.001).

The morphology of mock-inoculated and ASFV-infected MDMs was further compared by visualization of the actin cytoskeleton using Phalloidin-iFluor 594. Although the majority of cells exhibited a spherical morphology in both groups, a subset of MDMs displayed one or more straight, thin F-actin protrusions that extended beyond the size of the cell body from which they originated, resembling the description of TNTs (Figure 2A). A total of 600 p72-positive cells were examined for the presence of such protrusions at 9, 15, and 21 hpi in the ASFV-infected group (Figure 2B). Since no p72-positive cells were observed in the ASFV-infected group at 1.5 hpi or in the mock-inoculated groups at any of the timepoints, 600 randomly selected cells were similarly scored under these conditions to provide a comparative overview.

Very few cells exhibited protrusions at 1.5 hpi, with an average of one cell in the mock-inoculated group (± 1) and one cell (± 1.73) in the ASFV-infected group. Similar results were observed for the mock-inoculated group at 9, 15, and 21 hpi (2 ± 3.46, 1 ± 1.73, and 3.33 ± 3.05, respectively). In contrast, p72-positive cells presented a marked increase in the number of protrusions, with an average of 30 ± 23.80 cells exhibiting projections at 9 hpi (± 23.80) and 28.67 ± 11.93 cells at 15 hpi. A continued increase was further observed at 21 hpi, with 61.33 ± 29.4 cells displaying projections. Overall, ASFV infection of MDMs resulted in a significantly greater number of cells with long cellular protrusions, leading to statistically significant differences between ASFV-infected and mock-infected cells at 15 hpi (*p* < 0.01) and 21 hpi (*p* < 0.001).

In addition to these quantitative differences between the two groups, phenotypical differences were also observed. A well-pronounced vesicle-shaped CS at the distal end of the TNT-like projections in p72-positive cells was often clearly visible, whereas such a structure was absent in mock-inoculated cells with actin projections. To confirm that these structures were not exclusive to BE18, MDMs were infected with Est, E70, and NHV at an MOI of 1, following the described method. DNA, F-actin and the viral P72 antigen were visualized in cells fixed at 18 hpi. Similar to BE18-infected MDMs, TNT-like projections ending in the CS were also observed in MDMs infected with other ASFV isolates (Additional file 2). On the basis of this unique phenotype and the observation of projections in MDMs infected with other ASFV isolates, we designated these cells with this morphology as ASFV-induced segmented cells. These are defined as cells divided into two or more cell segments linked by thin and straight F-actin-containing tethers that are longer than one cell body (> 10 µm).

**Figure 3:**
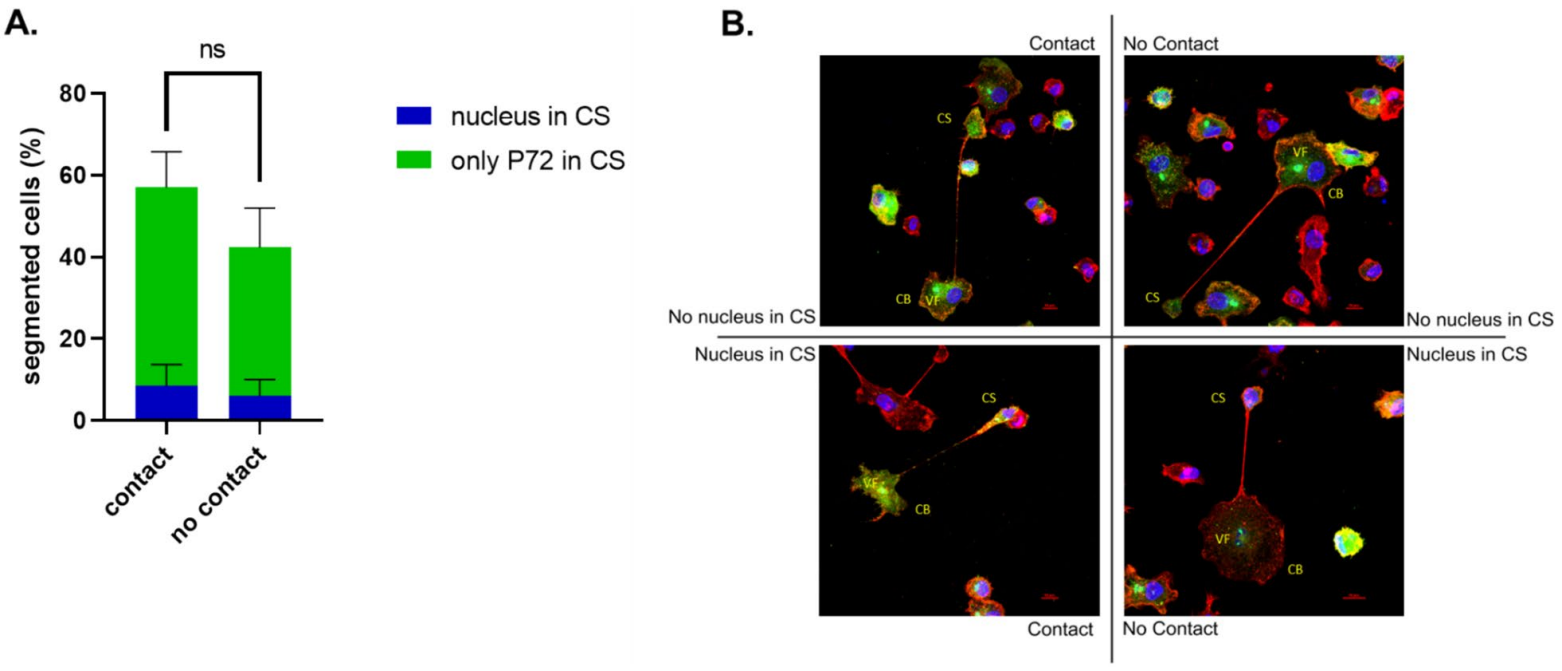
**ASFV-induced segmented cells can make contact with distant cells through the TNT-connected cell segment (CS), which, in certain cases, contains the cell nucleus. A** At 18 hpi, 300 segmented cells were scored for the presence or absence of contact with distant cells and for the presence or absence of the cell nucleus in the CS. **B** Illustrative images of p72-positive cells (green) with F-actin-containing projections (red) at 18 hpi. Different configurations are presented on the basis of the presence or absence of contact with distant cells and the presence or absence of the cell nucleus (blue) in the CS. Scale bar: 10 µm.

In our observations, the few mock-infected cells with TNT-like projections were always in contact with distant cells, whereas in the ASFV-infected group, only 57.6% (± 12.38) of segmented cells made contact with distant cells via this CS. Additionally, in 8.52% (± 5.19) of the contacting cells and 5.97% (± 4.07) of the non-contacting cells, the nucleus was not observed in the cell body (CB) but within the vesicle-shaped CS. The viral factory (VF), visualized by accumulated viral DNA and p72 antigens, which are typically positioned next to the nucleus in ASFV-infected cells [54], remained present in the remaining CB, even when the nucleus was observed in the CS (Figure 3).

In our experiments, TNT-like projections were observed in MDMs matured in the presence of hM-CSF, as well as in monocytes that were left to mature for 3 days without hM-CSF (data not shown). In both cases, TNT-like projections were observed with a similar phenotype, despite the significantly reduced number of adhering cells in the absence of hM-CSF.

### ASFV-induced segmented cells are highly heterogeneous in terms of the length of the TNTs and size of the cell segments

**Figure 4:**
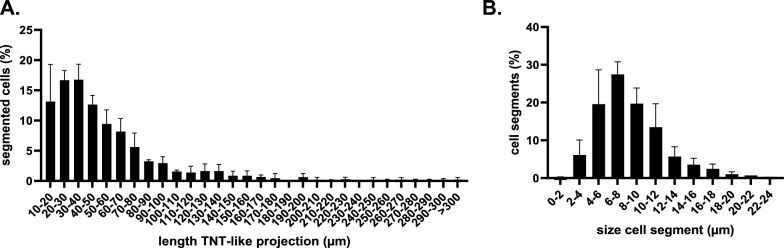
**High heterogeneity in the length of TNTs and size of the cell segments in ASFV-infected segmented cells was detected at 18 hpi. A** The measured lengths of the TNTs varied from a minimum of 10 µm to over 300 µm (*n* = 2), with the most commonly observed length ranging between 30 and 40 µm (16.77% ± 2.55). **B** A similar distribution pattern was observed for the diameter of the CS (*n* = 300), which ranged from just under 2 µm to 22–24 µm (*n* = 3). The most common diameter ranged between 6 and 8 µm (27.44% ± 3.34).

Next, the length of the TNTs and the diameter of the CS were measured and quantified from images acquired from 300 ASFV-induced segmented cells per animal (Figure 4). A high degree of heterogeneity was observed in the length of the TNTs, ranging from 10 µm to over 300 µm in extreme cases. The largest fraction of segmented cells had TNT lengths between 30 and 40 µm (16.77% ± 2.55).

Similarly, the diameter of the CS showed a high degree of variability, with the smallest measured diameter just under 2 µm and the largest between 22 and 24 µm. Most CSs had diameters ranging from 6 to 8 µm (27.44% ± 3.34). In most cases, the CS can be distinguished from the CB by its vesicle-shaped morphology, smaller size, absence of the nucleus, or a combination of these characteristics.

### ASFV induces TNT-like projections that do not contact the substrate

**Figure 5:**
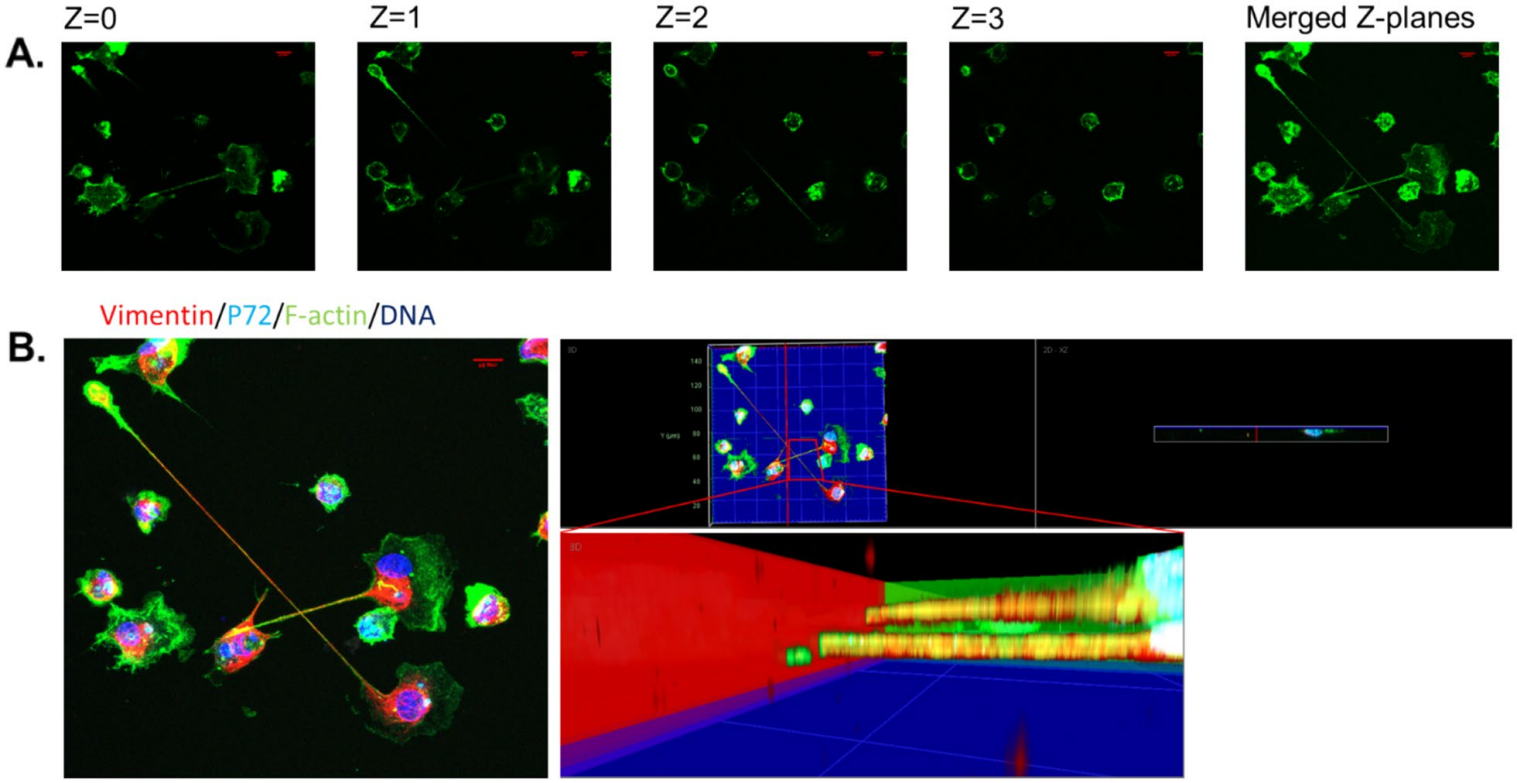
**ASFV-induced projections are TNT-like projections**. **A** Representative confocal images of cell projections in ASFV-infected MDMs at 18 hpi, showing filamentous F-actin (green) after staining with phalloidin-iF 488 at different z-planes (z = 0–3). Both the cell segment (CS) and the cell body (CB) can be observed attached to the coverslip (z = 0). Fragments of the TNT-like projection can be seen in higher z-planes (z = 1 and z = 2, respectively) and in the merged z-planes after image acquisition. **B** Orthogonal projection of the same image presented in panel A, showing the presence of vimentin (red) with an anti-vimentin antibody (VI10), p72 (cyan) with anti-p72 (1BC11), F-actin (green) with phalloidin-iF 488, and nuclei (blue) with Hoechst 33342. Three-dimensional reconstruction and close-up visualization at the interface of both cellular projections confirmed that the projections cross each other without establishing contact with one another or with the surface (blue plane) of the coverslip on which the cells are seeded. Scale bar: 10 µm.

Confocal imaging was used to investigate the ASFV-induced TNT-like projections by reconstructing serial images taken at different Z-planes (1 µm intervals). This confirmed that, in addition to containing filamentous (F-)actin, the membrane tethers induced by ASFV infection of MDMs are not connected to the substrate, which is in line with the general definition of TNTs [55]. Hence, we termed these projections TNT-like projections (Figure 5A). Additionally, this feature of TNTs was further supported by the observation that straight TNT-like projections crossed each other without making contact (Figure 5B). As with the CB, the CS at the proximal end of the TNT made contact with the substrate, causing both the CS and CB to be focused in the same Z-plane when viewed with an inverted fluorescence microscope.

### The cell segment can be discriminated from the cell body on the basis of the polarized distribution of the cytoskeletal filaments

**Figure 6:**
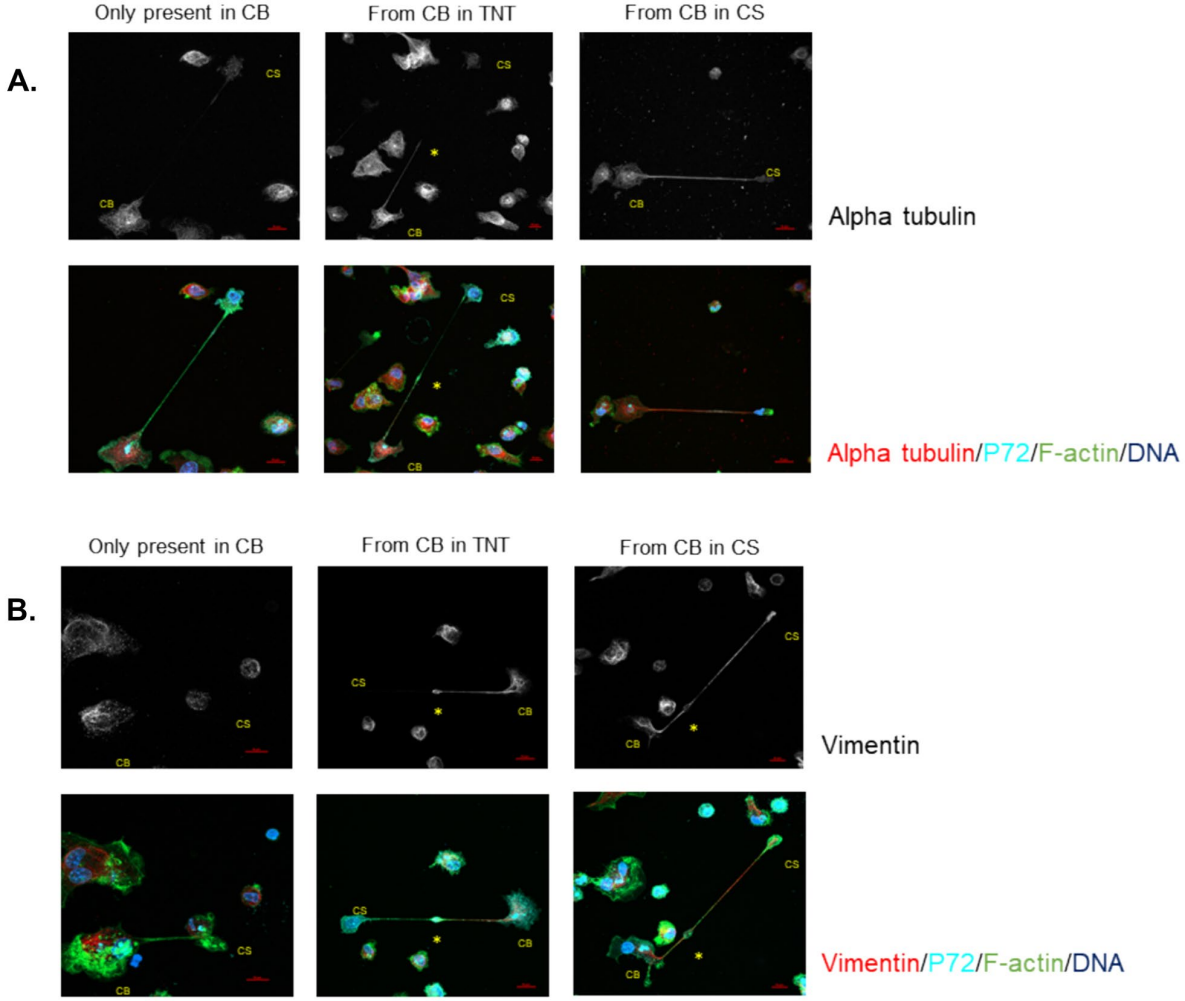
**Illustrative confocal IF images of the three different phenotypes of segmented cells after image acquisition**. **A** At 18 hpi, ASFV-infected MDMs were visualized for the presence of alpha-tubulin (red), p72 (cyan), F-actin (green), and DNA (blue) by IF staining. The upper row shows grayscale images of alpha-tubulin, clearly illustrating the different distributions of the three phenotypes. In the first phenotype, alpha-tubulin is present only in the cell body (CB) (left column). In the second phenotype, alpha-tubulin filaments extend from the CB into the TNT-like projection (middle column). In the third phenotype, alpha-tubulin extends from the CB into the cell segment (CS) (right column). When filamentous alpha-tubulin did not reach the CS (left and middle columns), aggregates of alpha-tubulin were observed in the CS. **B** ASFV-infected MDMs were visualized for the presence of vimentin (red), p72 (cyan), F-actin (green), and DNA (blue) by IF staining. The upper row shows grayscale images of vimentin, clearly illustrating the different distributions among the three phenotypes, similar to what was observed for alpha-tubulin. In certain cases, a spindle-shaped midbody (asterisk) was observed along the course of the TNT-like projection. Scale bar: 10 µm.

Visualization of other cytoskeletal components, in addition to F-actin, revealed a polarized distribution of these filaments (Figure 6). Microtubules and intermediate filaments, visualized using antibodies against alpha-tubulin and vimentin, respectively, seem to be involved in the architecture of these segmented cells. Three distinct phenotypes could be identified: (i) segmented cells where these cytoskeletal filaments are only observed in the CB, (ii) cells where the filaments extend from the CB into the lumen of the TNT, and (iii) cells where these filaments reach the lumen of the CS (Figures 6A and B).

**Figure 7:**
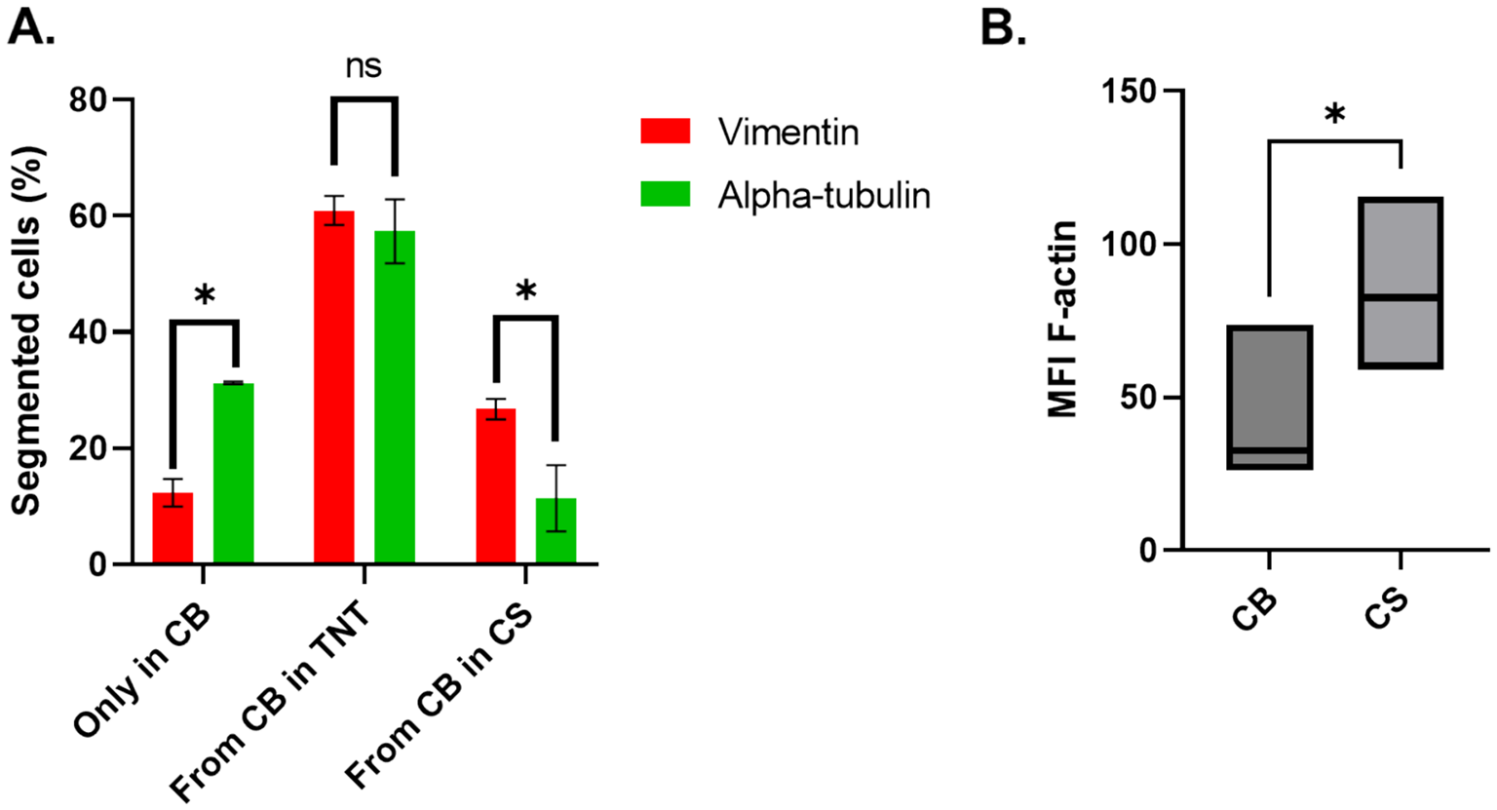
**Distribution of cytoskeletal filaments in ASFV-segmented cells. A** At 18 hpi, a total of 180 segmented cells were scored for the intracellular distribution of vimentin or alpha-tubulin after confocal image acquisition. Significantly more segmented MDMs exhibited alpha-tubulin, which was present only in the cell body (CB) (*p* < 0.01). The largest fraction of segmented MDMs showed vimentin or alpha-tubulin extending from the CB into the TNT-like projection. Significantly more cells presented with vimentin present in the CS than with filamentous alpha-tubulin extending into the CS (*p* < 0.05). **B** The mean fluorescence intensity (MFI) of F-actin in 180 cell segments (CSs) and their corresponding cell bodies (CBs) were determined after confocal imaging. The average MFI in the CSs was almost double that observed in the CB (*p* < 0.01).

Significantly more segmented cells (31.25% ± 0.24, *p* < 0.01) expressed alpha-tubulin, which was present exclusively in the cell body (CB), than vimentin, which was present exclusively in the CB (12.34% ± 2.35) (Figure 7A). The largest fraction of segmented cells showed polymerized vimentin and alpha-tubulin extending from the CB into the TNT but not reaching the lumen of the cell segment (CS) in 60.92% (± 2.48) and 57.34% (± 5.5) of the cases, respectively. Finally, significantly more vimentin filaments (26.74% ± 1.74, *p* < 0.05) were observed extending from the CB into the CS than alpha-tubulin filaments (11.42% ± 5.69), although the organization of both filaments in the CS was always lower than that in the CB. When filamentous alpha-tubulin did not extend into the CS, aggregates were observed in this segment.

These findings contrast with the presence of F-actin, which was always present in all compartments of the segmented cells. However, a significantly greater mean fluorescence intensity (MFI) of F-actin was observed in the CS (85.77 ± 28.47, *p* < 0.01) than in the CB (44.11 ± 25.49) (Figure 7B).

### The TNT-connected cell segments contain various cellular organelles, vesicular material, and free virions, likely originating from the perifactorial cytosolic compartment

**Figure 8:**
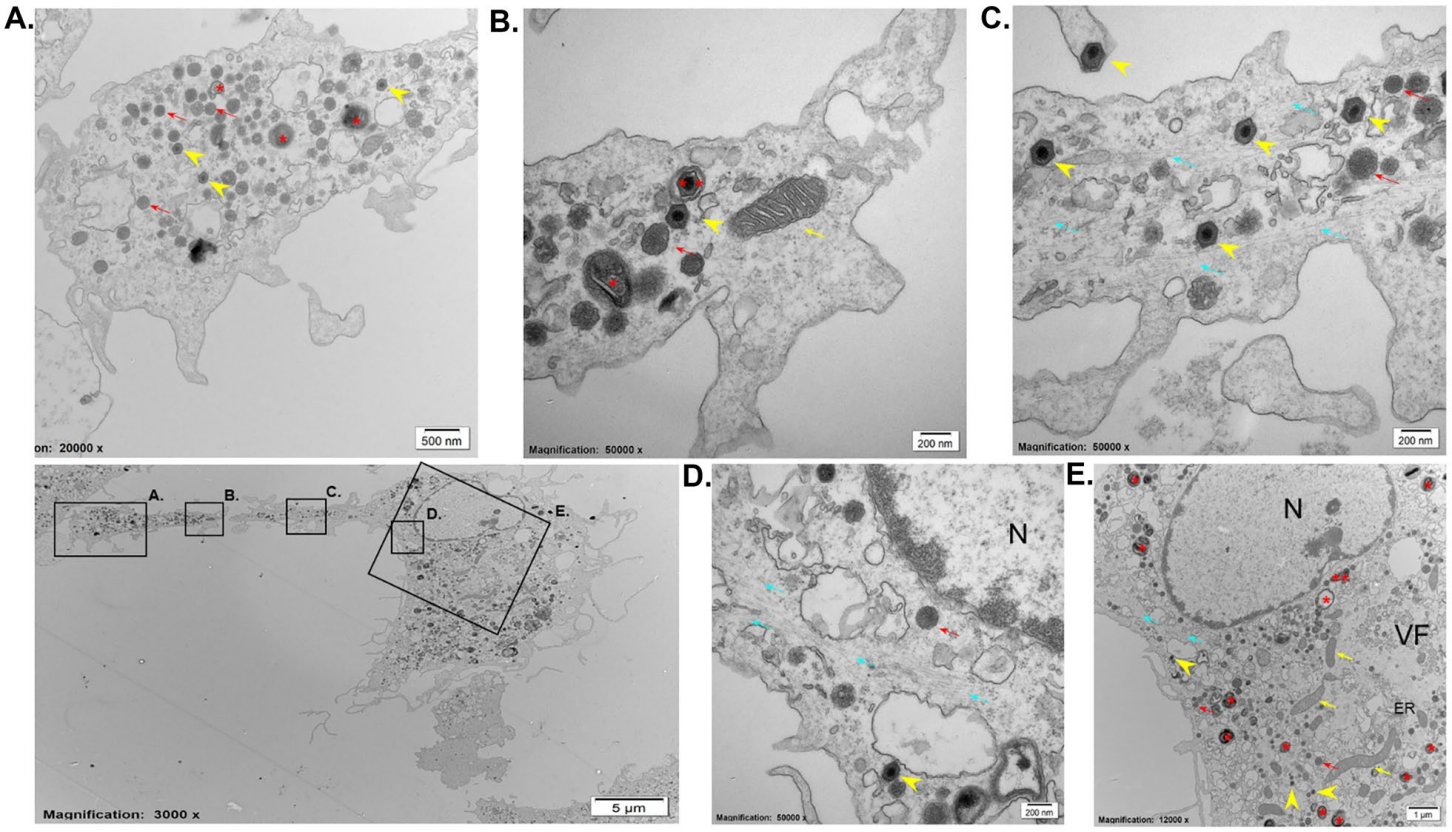
**Transmission electron microscopy image of an ASFV-segmented cell at 18 hpi**. **A**, **B** Close-up visualization of the cell segment revealed the presence of lysosomes (red arrows), fully assembled virions (yellow arrowheads), vesiculated cellular material (red asterisk), vesiculated viral material (double red asterisks), and mitochondria (yellow arrows). **C** High-magnification view of the TNT-like projection showing bundled cytoskeletal filaments (blue arrows), fully assembled virions (yellow arrowheads), and lysosomes (red arrows). **D**, **E** Close-up of the base of the TNT-like projection demonstrating the presence of assembled virions (yellow arrowheads), lysosomes (red arrows), and bundled filaments extending from the VF along the nucleus towards the CS (blue arrows).

To further characterize the contents of all the compartments in AFSV-induced segmented cells, transmission electron microscopy (TEM) was performed (Figure 8). Ultrathin sections (70 nm) were successfully prepared, crossing the entirety of the segmented cells in a limited number of cases (*n* = 6), just above the seeding surface. In all instances, the TNT-like projections connecting the two segments could be distinguished from other membrane projections by the presence of bundled cytoskeletal filaments within the lumen of the projections. These bundles extended from the VF and could be traced towards the base of the CS, suggesting that these bundles are at least partially composed of vimentin.

Along the length of the TNT, bundles of filaments were interspersed with elongated or damaged mitochondria as well as viral and cellular components, some of which were vesiculated. In all the examined cases (*n* = 6), the CS was filled with partially and fully assembled virions, lysosomes, vesicular material of both cellular and viral origin and mitochondria. The presence and alignment of bundled cytoskeletal filaments suggest that the contents of the CS are likely derived from the region surrounding the VF in a manner similar to the nucleus observed in a subset of segmented cells (Figure 8A).

**Figure 9:**
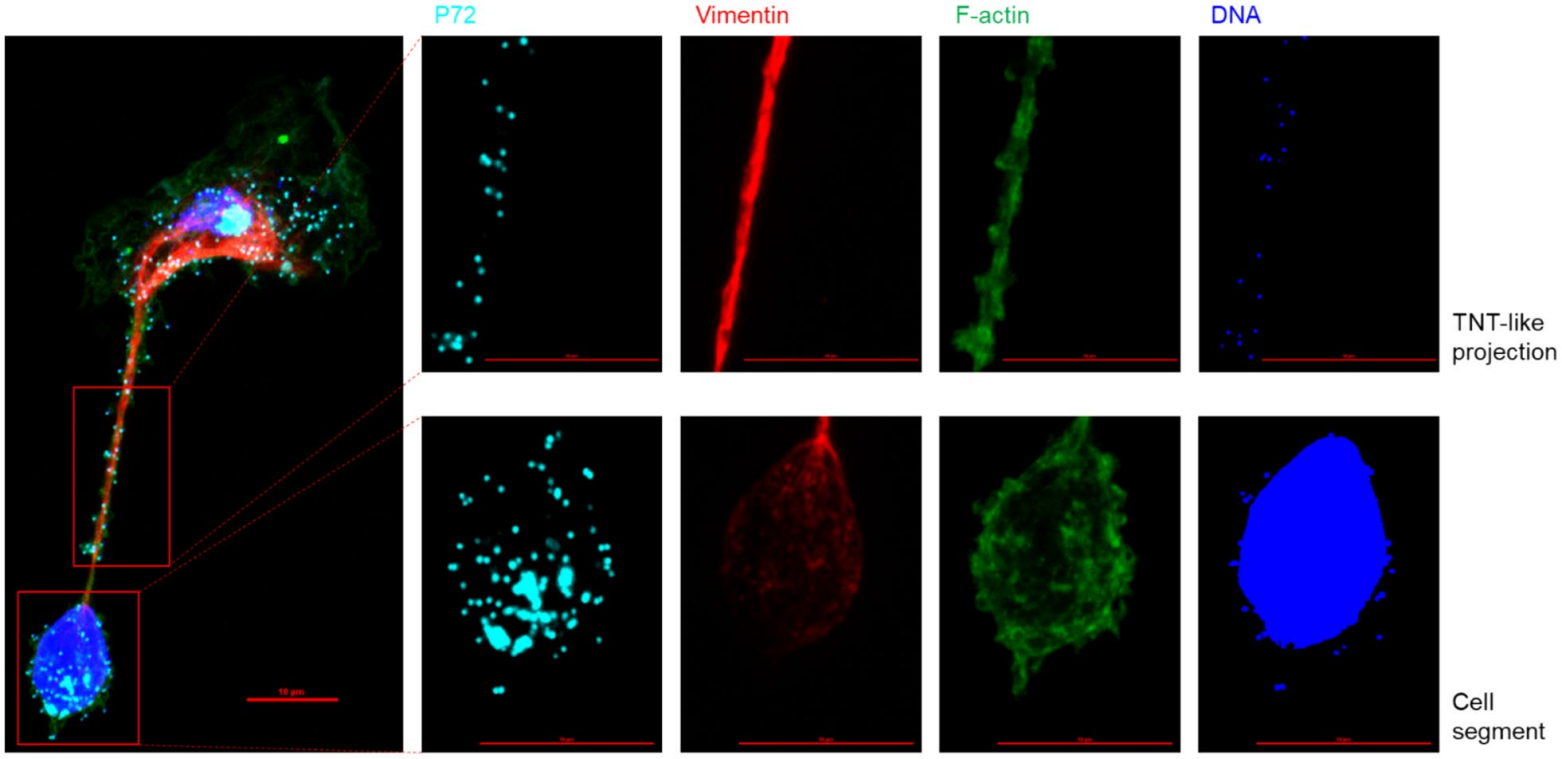
**Double-positive ASFV virions (p72 + DNA +) colocalized with small actin-rich protrusions on the TNT-like projection surface and within the cell segment of an ASFV-segmented cell at 18 hpi.** The upper row shows double-positive virions (p72 + DNA +) localized at small actin-rich protrusions on the TNT-like projection surface, whereas the bottom row highlights similar structures within the cell segment. The cytoskeleton was stained for vimentin (red), F-actin (green), cellular and viral DNA (blue), and the viral p72 antigen (cyan). Scale bar: 10 µm.

Furthermore, fully assembled virions were observed at the tips of small membrane protrusions along the TNT-like projections (e.g., the upper virion in Figure 8C). Thus, fully assembled virions were present in the CB, the TNT-like projections, and the CS. This finding is further supported by the double-positive labelling of p72 and DNA puncta, corresponding to the size of fully assembled virions detected in all segments of these segmented cells by confocal microscopy (Figure 9). In the TNT-like projections and the CS, most of these puncta were observed near the surface or at the tips of small actin-positive protrusions.

## Discussion

In this study, we demonstrated that African swine fever virus (ASFV) infection of porcine monocyte-derived macrophages (MDMs) triggers the formation of cellular protrusions. In addition to F-actin, which was consistently observed, microtubules and vimentin were also present in these projections, with vimentin being particularly abundant. Using confocal imaging, we observed that these protrusions did not make contact with the substrate, which, together with the presence of F-actin, is a defining characteristic of tunneling nanotubes (TNTs) [55]. A remarkable feature of the ASFV-induced protrusions was the presence of distinct vesicle-shaped cell segments at their tips, which made contact with the substrate. Electron microscopy further confirmed these observations and revealed that virions were present throughout the protrusions. These observations provide new insights into the cytopathic effect (CPE) induced by ASFV in its primary target cell type.

This study is limited to a single cell type and includes only a narrow validation of the described phenotype across four ASFV strains. Consequently, the broader relevance of this observation to ASFV infection remains uncertain. Further research is therefore needed to draw more general conclusions regarding ASFV-induced cell segmentation. Many viruses induce morphological changes during replication, leading to either host cell death or morphological alterations [56]. These changes serve as valuable diagnostic markers for detecting viral infections in cell monolayers, which is termed the cytopathic effect (CPE) in this context. CPEs can manifest in various forms, including cell lysis at the end of viral replication [57], syncytia formation [58], inclusion bodies [59], or different forms of cellular protrusions [37]. Since CPEs reflect diverse interactions between viruses and host cells, they provide valuable insights into these processes, helping to elucidate specific pathological events. The role of CPEs is complex, as they not only may facilitate viral replication [60] but also modulate host cellular functions, often offering clues to the clinical manifestations observed in infected individuals, as described with other viruses [61].

The CPE induced by ASFV in monocytes is characterized by cell detachment, enlargement, and rounding, which progressively results in the formation of grape-like clusters of 3–20 or more cells that eventually lyse [62]. Recent high-resolution observations have provided more specific details regarding ASFV-infected macrophages. Yuan et al. reported enhanced mitochondrial damage and subsequent autophagy, a reduced number of pseudopodia impairing phagocytosis, and a decrease in lysosome size and acidification, further suggesting that phagocytic activity is suppressed [63]. A distinctive feature of ASFV infection is the formation of filopodia-like projections in Max cells, porcine aortic endothelial cells and porcine bone marrow-derived macrophages [64]. Intracellular ASFV particles projected out of infected cells at the tips of these small protrusions, which consisted of parallel, unbranched actin filaments. The description of these filopodia-like projections is consistent with the smaller actin protrusions in our study in the CB, CS, and TNT-like projections. However, these protrusions differ from our observations of TNT-like structures in terms of quantity, dimensions and content.

A greater number of filopodia-like projections were observed by Jouvenet et al., each containing a single ASFV particle at its tip [64]. These projections were smaller in diameter than the virion itself, and their lengths did not exceed 10 µm in porcine bone marrow-derived macrophages, with F-actin as the sole cytoskeletal backbone. In contrast, we observed that only 1–3 TNT-like projections per infected cell were present, with lengths starting at 10 µm and extending up to 300 µm or more. These projections often contain all three major types of cytoskeletal filaments and end in well-pronounced, vesicle-shaped tips housing various cellular organelles and multiple virions. The size of these tips, or CS, ranged between 2 and 24 µm, comparable to the size of the remaining CB in extreme cases. Despite these differences, it is plausible that both types of projections may play complementary roles in viral spread. We hypothesize that ASFV virions may leave TNT-like projections via filopodia-like protrusions along the cell surface and may reach more distant cells. Furthermore, an accumulation of filopodia-like projections at the sites of cell-to-cell contact was observed by Jouvenet et al. [64]. Consequently, the formation of a TNT-like projection offers a means to establish cell-to-cell contact with more distant cells. This phenomenon, known as “virus surfing”, allows the virus to move along or just beneath the surface of cellular protrusions, facilitating entry into more distant cells. This mechanism is proposed to transfer the virus more efficiently than the cell-free route [65]. Live-cell imaging will be needed to confirm this potential mechanism.

Through confocal imaging, we observed that the ASFV-induced cellular protrusions did not make contact with the substrate, a characteristic feature of TNTs [38, 66]. TNTs can form through two distinct mechanisms: the cell dislodgement mechanism, where two initially contacting cells separate, and the protrusion elongation mechanism, where one cell extends a filopodia-like protrusion towards another [66]. In addition to hovering over the substrate, TNTs are defined by their ability to connect at least two cells and contain F-actin [66]. Since ASFV-segmented cells contacted distant cells in only 57.6% (± 12.38) of the cases, we infer that these cells likely formed via the protrusion elongation mechanism rather than the dislodgement mechanism. Alternatively, because the CS makes contact with the substrate, it is possible that this part of the cell actively migrated away from the main cell body while maintaining contact via the TNT-like projection. This phenomenon has been observed in neutrophils, where it is referred to as cytokineplasts [67] as well as in glioma cell invasiveness [68]. We denominate these ASFV-induced non-adherent protrusions as TNT-like projections.

In addition to TNTs, several other macrophage protrusions share similarities with our description. Owing to these resemblances, terminology is often used interchangeably when referring to these membranous extensions. Collectively, these protrusions are categorized as TNTs, cytonemes or retraction fibres [69]. Viruses can manipulate the host cell cytoskeleton to facilitate direct transmission from virus-producing cells to neighbouring cells [70]. One such strategy involves the induction of these cellular protrusions to bridge the extracellular space between susceptible cells.

Hu et al. [37] described cytonemes as specialized filopodia that establish direct contact with neighbouring cells, enabling the transfer of bioactive materials through their tips. Cytonemes are formed during retrovirus infections to increase cell-to-cell transmission and are composed of only F-actin [71]. This contrasts with our observations, where intermediate filaments were present in most TNT-like projections, alongside actin filaments and microtubules.

In the context of cytonemes, when the membrane tip fuses with the membrane of a distant cell, establishing cytosolic continuity, the protrusion is classified as a TNT [37]. TNTs are triggered by numerous viral infections to facilitate direct cell-to-cell transmission [39, 41]. However, in the case of ASFV, the cellular protrusion does not immediately contact a distant cell but instead leads to the formation of a distinct CS, which, in some cases, makes contact with neighbouring cells. Notably, our electron microscopy data did not reveal cytoplasmic or cytosolic continuity between the CS and the contact cell. The formation of these TNT-like projections alone does not fully explain the function or significance of this CS. Therefore, we speculate that these TNT-like projections provide ASFV with an additional advantage, possibly enhancing its dissemination between distant cells.

Additionally, migrating cells leave behind long tubular structures known as retraction fibres, which form a branched network of actin protrusions [72]. At the tips and intersections of these fibres, relatively large vesicles containing relatively small vesicles, called migrasomes, are generated [72]. Migrasomes, which are induced by Chikungunya virus, also mediate intercellular spread [73]. Retraction fibres differ from our observed protrusions in their branched morphology, cytoskeletal composition, and migrasome size, which do not exceed 3 µm [72]. These distinctions suggest that ASFV-induced segmented cells represent a unique feature of AFSV infection.

The cell nucleus was observed within the CS in 15.07% of the cases (± 1.04), leading to spatial separation of the viral factory (VF) from the host cell nucleus. While the precise role of the nucleus in ASFV replication remains unclear, a recent study in which DNA scope in situ hybridization was used to examine the subcellular localization of viral DNA during ASFV infection failed to detect viral DNA in the nucleus at any stage of the infection cycle [74]. Furthermore, since ASFV encodes its own set of enzymes required for DNA priming and synthesis, the involvement of a nuclear phase in ASFV DNA replication is unlikely. Both ASFV and the host cell rely on nucleotides synthesized in the cytosol for DNA replication [75], suggesting that ASFV may take advantage of the physical separation between the host nucleus and the cytoplasm to facilitate or optimize viral DNA replication. Further research is needed to clarify the functional significance of this spatial compartmentalization during ASFV infection. To our knowledge, the formation of segmented cells in the context of infectious diseases has not yet been documented. However, as previously mentioned, segmented cell formation has been observed during glioma cell invasion [68]. In malignant glioma brain tumor dissemination, anucleate cell fragments, termed microplasts, can spontaneously detach from the original glioma cells after initially remaining connected through a cellular protrusion. This process morphologically resembles ASFV-induced segmented cell formation. These fragments are motile and likely contribute to extracellular matrix invasion because their size is smaller than that of parent cells. Alternatively, these microplasts could help condition the microenvironment by secreting factors such as matrix metalloproteinases, facilitating tumor cell invasion. Given that the CS made contact with the substrate and, in some cases, resembled migrating macrophages, it is reasonable to assume that the CS actively migrates away from the remaining cell body (CB), possibly to promote virus dissemination, modulate the extracellular matrix, and/or enhance entry into distant cells. Furthermore, these microplasts ranged in size from 2% to nearly half of the area of their parent cells, which correlates with the sizes of the CSs observed in the ASFV-segmented cells. Further research using 3D matrix models could help elucidate this potential function.

Another process where cell segmentation occurs and where the cellular segments remain connected via a tunneling nanotube is during the formation of exophers [43]. Exophers are observed in neurodegenerative and prion diseases [43, 76] and are formed as a cellular response to proteotoxic stress, expelling toxic cargo when proteostasis pathways, such as the ubiquitin‒proteasome and autophagy systems, are overwhelmed [64]. ASFV encodes proteins capable of inhibiting both of these pathways [77, 78]. Furthermore, the formation of aggresomes—cellular structures that sequester misfolded proteins in intermediate filament cages near the nucleus when proteostasis mechanisms are overwhelmed [79]—appears to trigger exopher formation [76]. Heath et al. described ASFV-induced viral factories as sharing similarities with aggresomes, further supporting this hypothesis [54]. In this context, all cytoskeletal filaments seem to play specific roles in exopher formation [76], which could explain their presence in ASFV-segmented cells.

Despite these similarities, substantial differences and the lack of irrefutable evidence prevent us from definitively claiming that our observations suggest exopher biogenesis in ASFV-infected MDMs. Although exophers have been observed in various cell types across different mammalian species, including humans, they have never been reported in macrophages. Additionally, the release of neurotoxic contents within exophers appears to benefit the producing neurons, as exopher formation has been shown to enhance neuronal function [43]. Therefore, it is logical that the cell nucleus is absent from exophers, whereas other organelles, such as damaged mitochondria and lysosomes, are commonly present [43]. While exophers have a relatively uniform diameter of approximately 4 µm [43], the diameter of our CS varies extensively, with most cases measuring between 6 and 8 µm (27.44% ± 3.34). We also observed irregularly shaped CSs, which do not match the typical ‘balloon-shaped’ morphology of exophers [43]. This could again be due to the migratory capacity of the macrophages after they contacted the substrate, as stated before.

In this study, we reported that ASFV infection induces the formation of elongated cellular protrusions in porcine monocyte-derived macrophages (MDMs), characterized by the presence of F-actin, microtubules, and an abundance of vimentin. A unique feature of these ASFV-induced structures is the formation of vesicle-shaped and ASFV-containing segments at their tips. This observation provides novel insights into ASFV-induced CPEs in its primary target cell type and may represent a specialized mechanism facilitating viral spread or enhancing virus replication. More research is needed to further elucidate the functions and biogenesis of these ASFV-segmented cells, keeping in mind their potential implications in the pathogenesis of ASFV.

## Supplementary Information

**Additional file 1. Infection of monocyte-derived macrophages with ASFV BE18 (MOI=1). (A)** At the indicated time points, the supernatants were collected, and the ASFV copy number was determined via RT‒qPCR. (**B**) Additional supernatant samples were collected to determine the infectious virus titre via an immunoperoxidase test (IPT).

**Additional file 2. Representative images of MDMs infected with different strains of ASFV (MOI=1).** At 18 hpi, the cells were fixed and visualized for the presence of viral p72 (green) using a mouse monoclonal antibody (1BC11), F-actin (red) with Phalloidin-iFluor 594, and DNA with Hoechst 33342. Long, straight TNT-like projections (yellow arrows) were observed in MDMs infected with Belgium 2018/01 (BE18), E70, Est15/WB-Valga-6 (Est) or NH/P68 (NHV). Scale bar: 10 µm.

## Data Availability

The data that support the findings of this study are available upon reasonable request from the authors.
