# Supplementary figures and images for: African swine fever virus infection of porcine peripheral blood monocyte-derived macrophages induces the formation of tunneling nanotube-connected large vesicle-like cell segments: a potential mechanism for intercellular ASFV trafficking
Source: Vet Res. 2025 Jul 10;56:148. doi: 10.1186/s13567-025-01582-0 (PMC12247315; doi:10.1186/s13567-025-01582-0)

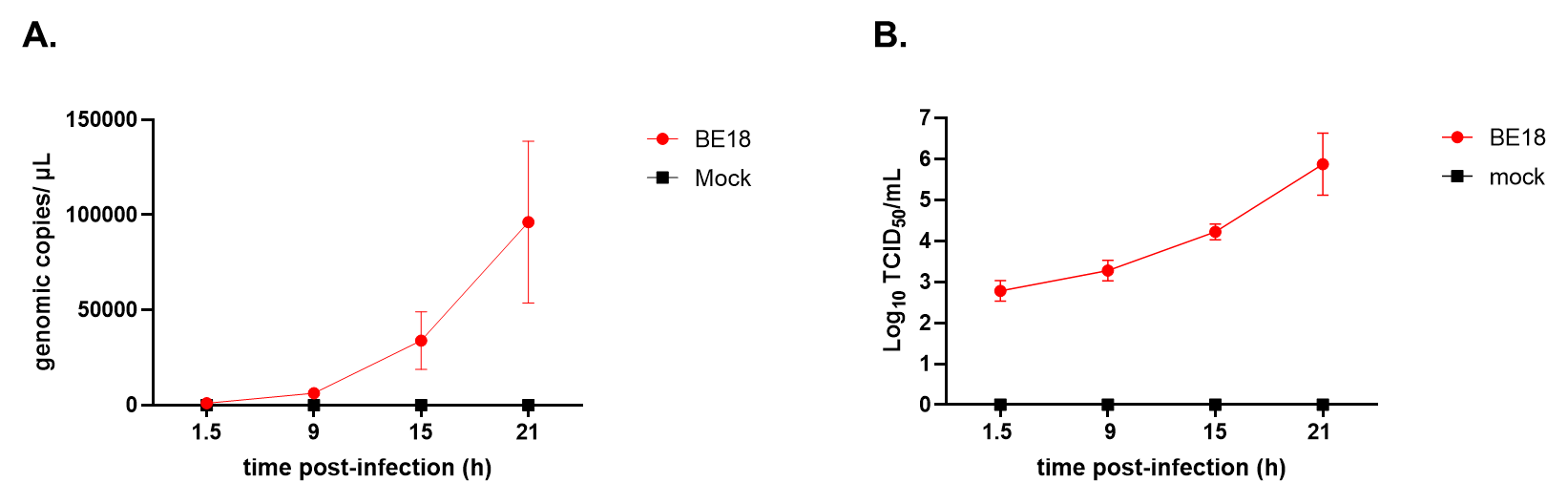


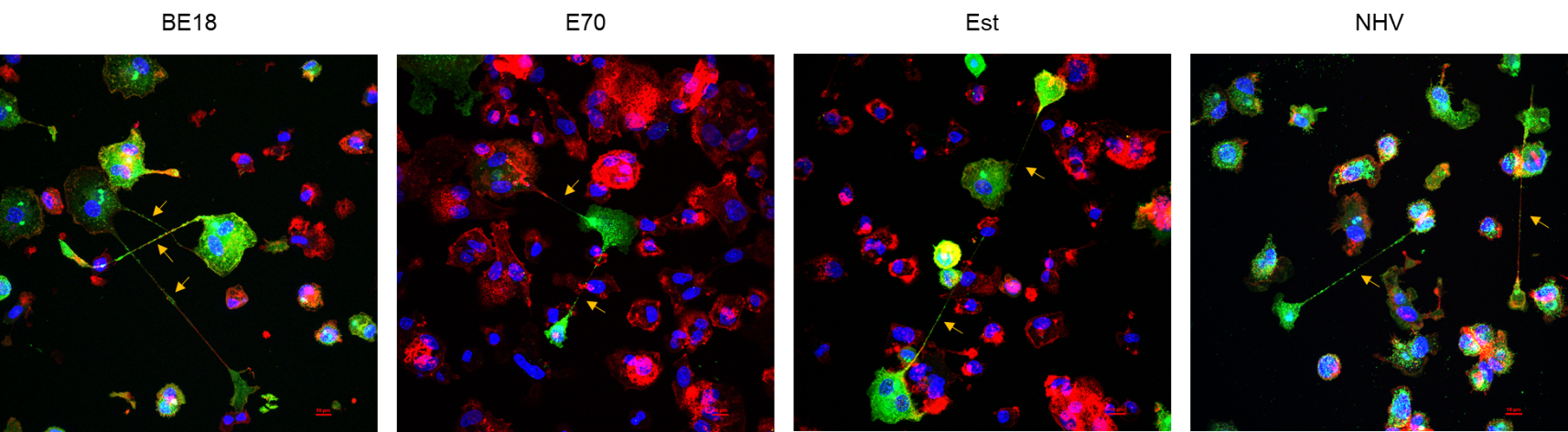

Supplement: Supplementary file 1 — Additional file 1. Infection of monocyte-derived macrophages with ASFV BE18 (MOI=1). (A) At the indicated time points, the supernatants were collected, and the ASFV copy number was determined via RT‒qPCR. (B) Additional supernatant samples were collected to determine the infectious virus titre via an immunoperoxidase test (IPT). [file 13567_2025_1582_MOESM1_ESM.docx]

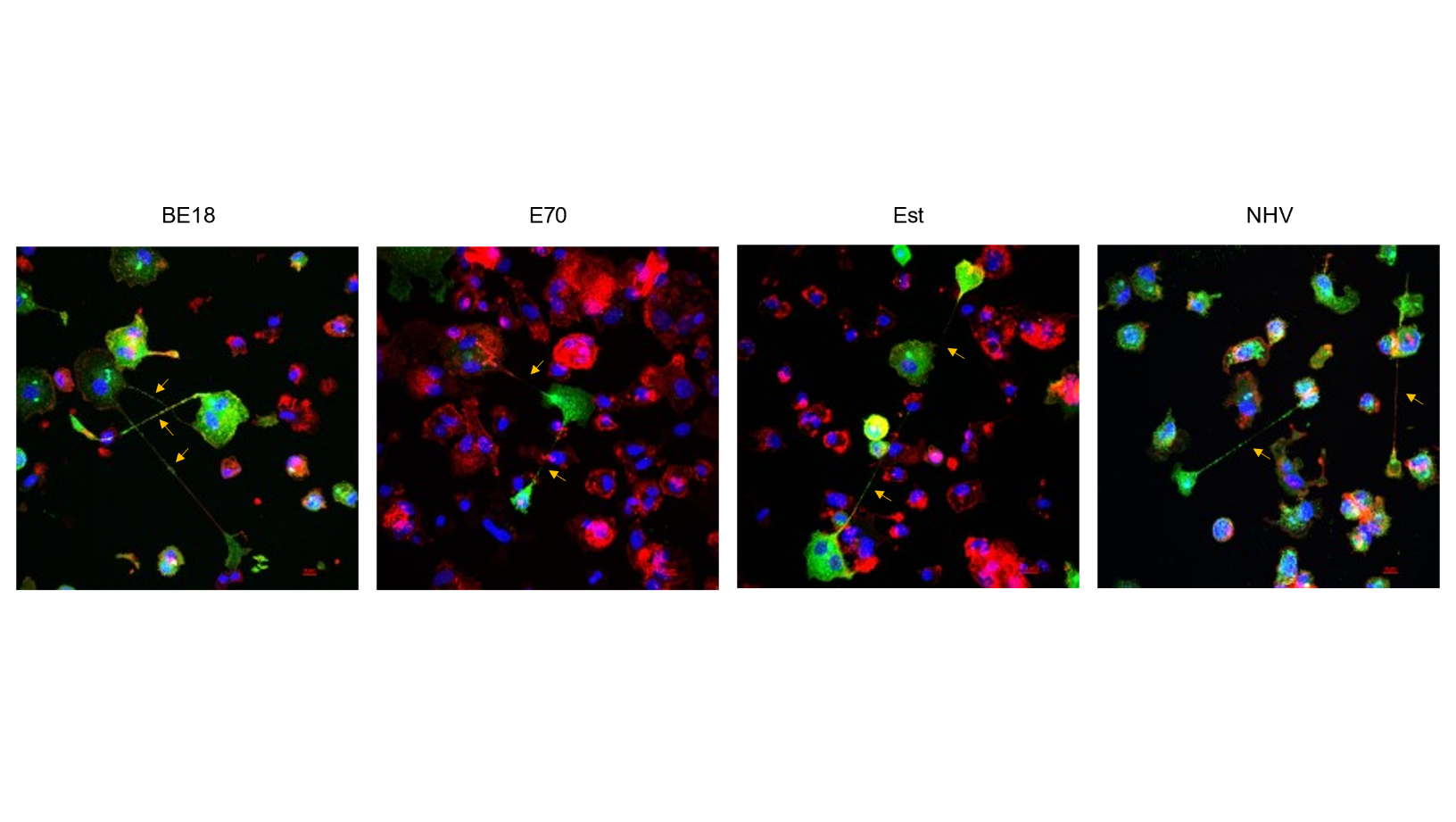

Supplement: Supplementary file 2 — Additional file 2. Representative images of MDMs infected with different strains of ASFV (MOI=1). At 18 hpi, the cells were fixed and visualized for the presence of viral p72 (green) using a mouse monoclonal antibody (1BC11), F-actin (red) with Phalloidin-iFluor 594, and DNA with Hoechst 33342. Long, straight TNT-like projections (yellow arrows) were observed in MDMs infected with Belgium 2018/01 (BE18), E70, Est15/WB-Valga-6 (Est) or NH/P68 (NHV). Scale bar: 10 µm. [file 13567_2025_1582_MOESM2_ESM.docx]
